# The Principles and Practice of Hydrotherapy

**Published:** 1900-01

**Authors:** 


### Books and Magazines.

THE PRINCIPLES AND PRACTICE OF HYDROTHERAPY. A Guide to the Application of Water in Disease. For Students and Practitioners of Medicine. By Simon Baruch, M. D., Consulting Physician to the Montefiore Home for Chronic Invalids, Etc., New York. With numerous illustrations. 435 pages. Price, $4.00 net. New York: Wm. Wood & Co. 1898.

In this book water is discussed as a therapeutic agent in the same way other agents are discussed in regular text-books on therapeutics. The work is divided into twenty chapters, and consists in the first seventy pages of a discussion of the physiological effects of water, the rationale of its action in health, its effects upon the distribution of the blood and its effects on the composition of the blood, on the respiration, muscular system, and on the temperature. The next ninety or hundred pages are devoted to the practice of hydrotherapy, including ablution, half-bath, affusion, sheet bath, cold bath, wet pack, wet compress, and precordial compress. The next hundred or more pages are devoted to the full bath, its indication and contra-indication in fevers, objection to the Brand method, etc. The douche, irrigation, lavage, enteroclysis, heating and cooling internal parts, the therapeutic uses of steam, etc., are given ample discussion.

The author in his application of the various uses of water to disease is strongest, and shows that he is a broad liberal thinker, and, above all, a man of large clinical experience. In other words, he seems to be a general practitioner, for no specialist in this line ever gave such strong evidence of being thoroughly practical.

The book is heartily commended and is timely.

